# Microneedling for Non-cosmetic Dermatologic Conditions: A Systematic Review of Efficacy and Safety

**DOI:** 10.7759/cureus.90857

**Published:** 2025-08-24

**Authors:** Amany Mashi, Rayim A Oraybi, Manar S Hakami, Abdulrahman M Altalhi, Wafa A Alrezqi, Taif K Hakami, Alanoud M Masmali, Asma F Alshahrani, Raghad A Alamri, Alanoud A Alanazi

**Affiliations:** 1 Dermatology, Armed Forces Hospital, Jazan, SAU; 2 College of Medicine, Jazan University, Jazan, SAU; 3 College of Medicine, King Abdulaziz University, Jeddah, SAU; 4 College of Medicine, Al Baha University, Al Baha, SAU; 5 College of Medicine, Bisha University, Bisha, SAU; 6 College of Medicine, King Khalid University, Abha, SAU; 7 College of Medicine, Al-Jouf University, Al-Jouf, SAU

**Keywords:** acne vulgaris, actinic keratoses, adjunct therapy, hyperhidrosis, melasma, microneedling, non-cosmetic dermatology, randomized controlled trials, systematic review, vitiligo

## Abstract

Microneedling, a minimally invasive dermatologic procedure, has expanded beyond cosmetic use to treat conditions such as vitiligo, hyperhidrosis, melasma, acne vulgaris, and actinic keratoses. This systematic review, conducted in accordance with PRISMA guidelines, included 15 randomized controlled trials involving approximately 1,200 participants identified through searches of PubMed, Web of Science, Scopus, and Cochrane Central Register of Controlled Trials (CENTRAL) up to July 2025. Across multiple conditions, microneedling - particularly when combined with adjunct therapies - demonstrated significant clinical benefits. In vitiligo, microneedling with tacrolimus or 5-fluorouracil (5-FU) achieved repigmentation rates of 40-76.6% compared with 16.9-39.9% for monotherapy. In hyperhidrosis, fractional microneedle radiofrequency (FMR) reduced sweat production, although botulinum toxin A yielded longer-lasting results and greater patient satisfaction. For melasma, combinations with tranexamic acid or cysteamine produced notable modified melasma area and severity index (mMASI) score reductions (29-50%), while in acne vulgaris, microneedle radiofrequency (MRF) outperformed photodynamic therapy (PDT), achieving an 81% reduction in inflammatory lesions versus 73%, with fewer adverse events. Microneedling-assisted PDT also improved actinic keratoses clearance rates by 18% (76% vs 58%). Adverse effects such as transient erythema and mild pain were consistently reported but generally well tolerated. Overall, current evidence supports microneedling as an effective and safe treatment for diverse non-cosmetic dermatologic conditions, particularly when integrated with topical or procedural adjuncts, though standardized protocols and long-term outcome data remain needed.

## Introduction and background

Microneedling, also known as collagen induction therapy, has gained traction in dermatology as a minimally invasive procedure that stimulates dermal remodeling through controlled microinjuries [1,2]. Rooted in the principle of percutaneous collagen induction, it promotes collagen and elastin production, contributing to improved skin texture and scar reduction [1,2].

Historically, microneedling has been widely applied for cosmetic purposes such as acne scar reduction, skin rejuvenation, and fine line improvement [2,3]. More recent literature has expanded its use into clinical dermatology, targeting conditions beyond aesthetics, including vitiligo, hyperhidrosis, melasma, actinic keratoses, and acne vulgaris [3,4].

The rationale for these non-cosmetic applications partly lies in their ability to enhance the transdermal penetration of topical agents via micropore channels [4,5]. Several studies have used microneedling as a drug delivery enhancer in combination with agents such as tacrolimus, pimecrolimus, 5-fluorouracil (5-FU), tranexamic acid, and photosensitizing agents for photodynamic therapy (PDT) [4,5].

Evidence from randomized controlled trials indicates that microneedling combined with topical therapies can significantly improve outcomes in conditions such as vitiligo and melasma compared to monotherapy [3,5]. Meta-analyses have confirmed enhanced efficacy and greater patient satisfaction with combined modalities over microneedling alone [3,6].

In the treatment of acne scars, microneedling monotherapy has demonstrated consistent benefits across multiple randomized trials, with significant improvements in objective scar grading and patient-reported satisfaction [3,6]. Notably, it has a low incidence of adverse effects such as post-inflammatory hyperpigmentation, making it suitable for darker skin types [3,6].

An emerging area of research involves microneedling with radiofrequency (RF) for conditions such as active acne and primary axillary hyperhidrosis. Fractional microneedle radiofrequency (FMR) has been shown to reduce inflammatory acne lesions more effectively than PDT, with fewer side effects such as post-inflammatory hyperpigmentation [4,7].

In hyperhidrosis, FMR has demonstrated the ability to thermally ablate sweat glands, with histologic evidence of glandular atrophy and reduced sweat output [4,8]. However, comparative trials suggest that botulinum toxin A may yield superior efficacy and patient satisfaction, particularly in long-term follow-up [8,9].

## Review

Methods

Search Strategy

**Figure 1:**
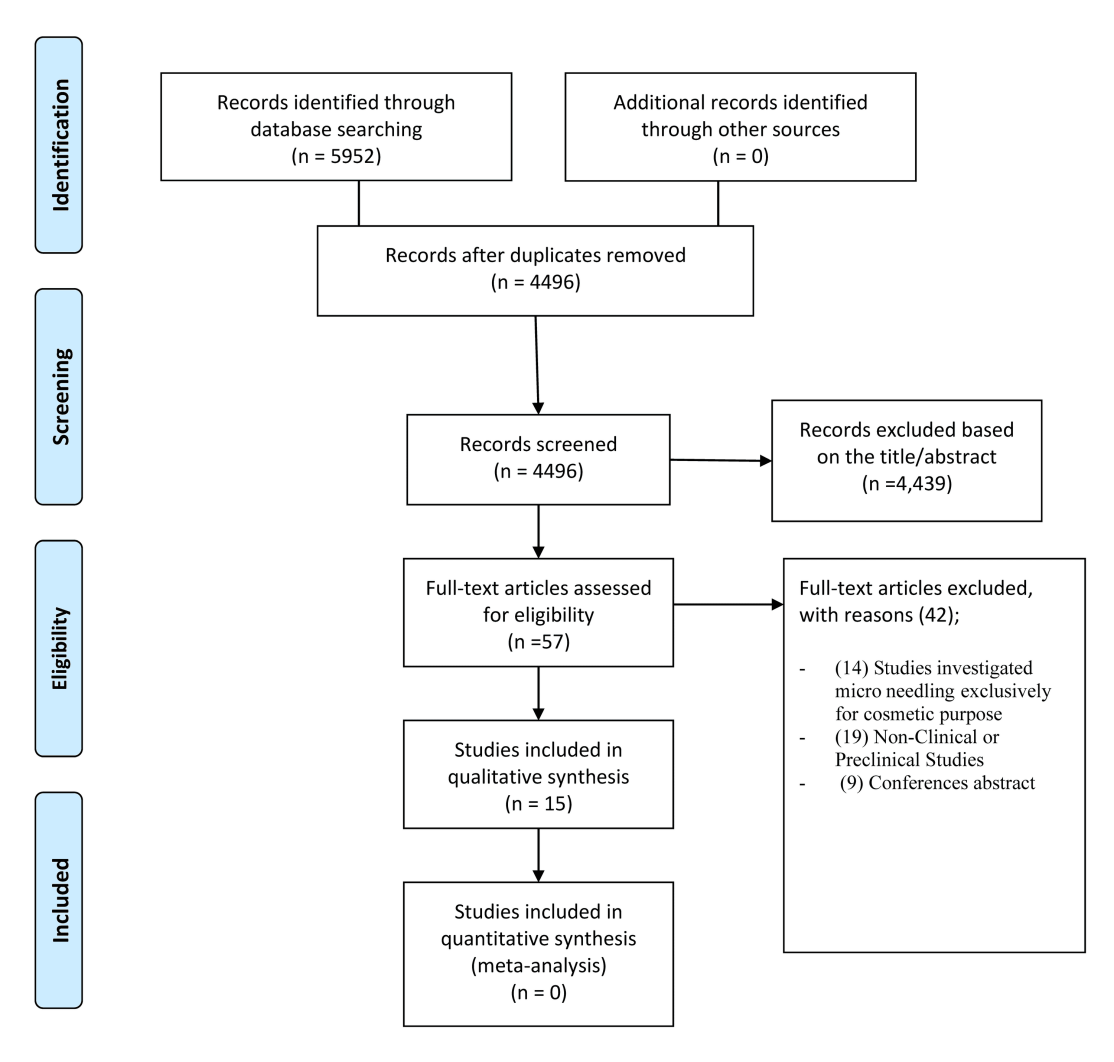
PRISMA flow diagram illustrating the study selection process PRISMA = Preferred Reporting Items for Systematic Reviews and Meta-Analyses

This systematic review was conducted and reported in accordance with the Preferred Reporting Items for Systematic Reviews and Meta-Analyses (PRISMA) guidelines [10]. A comprehensive search of PubMed, Web of Science, Scopus, and the Cochrane Central Register of Controlled Trials (CENTRAL) was performed from database inception through July 28, 2025. The search strategy combined free-text terms and controlled vocabulary, including “microneedling”, “collagen induction therapy”, “fractional microneedle radiofrequency”, along with targeted dermatologic conditions such as “vitiligo”, “melasma”, “acne”, “actinic keratosis”, “hyperhidrosis”, and “photoaging”. Boolean operators were applied to tailor the search for each database. Only English-language studies involving human participants were considered. Reference lists of included articles were also manually screened to identify additional eligible studies not captured in the initial search (Figure 1).

Eligibility Criteria

Eligibility criteria were defined according to the Population, Intervention, Comparison, Outcome, Study Design (PICOS) framework. We included English-language randomized clinical trials that: (i) enrolled patients with non-cosmetic dermatologic conditions such as vitiligo, acne, melasma, hyperhidrosis, actinic keratosis, or atrophic acne scars; (ii) used microneedling (with or without RF), either alone or in combination with topical or systemic therapies; (iii) included a comparator such as placebo, sham treatment, or other conventional therapies (e.g., botulinum toxin, laser, PDT); and (iv) reported clinical, patient-reported, or histological outcomes. We excluded observational studies, non-English publications, non-randomized trials, and studies available only as abstracts without accessible full-text articles.

Study Selection

Two reviewers independently screened the titles and abstracts of all retrieved articles against the eligibility criteria. Full texts of potentially relevant studies were then assessed to confirm inclusion. Discrepancies between reviewers were resolved through discussion with a third reviewer until consensus was reached.

Data Extraction

Data extraction was independently performed by two reviewers using a standardized, pre-defined form. Extracted items included study characteristics (author, year, country, design), participant demographics, target condition, intervention protocol (device type, needle depth, treatment frequency), adjunct therapy details (if applicable), comparators, outcome measures, key findings, adverse events, and author conclusions. Only data explicitly reported in the full text were included in the synthesis. Discrepancies were resolved through discussion or consultation with a third reviewer.

Quality Appraisal

The methodological quality of the included trials was independently assessed by two reviewers using the modified Downs and Black checklist for clinical trials [11], applied without further modifications. This 27-item tool evaluates four domains: reporting, external validity, internal validity, and power. Final scores classify studies as excellent (26-28), good (20-25), fair (15-19), or poor (≤14). Any disagreements were resolved through discussion until a consensus was reached.

Results

Study Selection

A total of 5,952 records were identified from PubMed (n = 418), Cochrane CENTRAL (n = 347), Scopus (n = 2,093), and Web of Science (n = 3,094). No additional records were retrieved through manual searches. After removing duplicates, 4,496 unique records remained; 4,439 were excluded after title/abstract screening. Full-text review was performed on 57 articles, excluding 41 (14 cosmetic microneedling, 19 non-clinical, nine abstracts). Fifteen studies [7,9,12-24] met all criteria and were included in the qualitative synthesis. No meta-analysis was performed due to unsuitable data. 

Study Characteristics

The 15 included studies, all randomized or controlled clinical trials, assessed microneedling for diverse dermatologic conditions. Most used split-face or split-body designs [9,15,17,23], while others used parallel-arm structures [16,19,22]. Participants ranged from 10 to 65+ years, mostly female (60-90%), with Fitzpatrick skin types III-IV predominating. Conditions included vitiligo, hyperhidrosis, acne (scarring and active lesions), melasma, photoaging, dry eye disease, and actinic keratoses (Table 1).

**Table 1:** Characteristics of included studies RCT = randomized controlled trial; FMR = fractional microneedle radiofrequency; Mn = microneedling; PDT = photodynamic therapy; ALA = aminolevulinic acid; TEWL = transepidermal water loss; VAS = visual analog scale; HDSS = hyperhidrosis disease severity scale; DLQI = dermatology life quality index; PAH = primary axillary hyperhidrosis; PRP = platelet-rich plasma; VASI = vitiligo area severity index; PGA = physician global assessment; BT-A = botulinum toxin A; IGA = investigator’s global assessment; MRF = microneedle radiofrequency; mMASI = modified melasma area and severity index; QoL = quality of life; OSDI = ocular surface disease index; SIT = Schirmer I test; BMA = biodegradable microneedle acupuncture; IDA = intradermal acupuncture; PIH = post-inflammatory hyperpigmentation; TA = tranexamic acid; GAIS = global aesthetic improvement scale; HMB-45 = human melanoma black 45 immunostaining marker; BL2, GB14, TE23, EX-HN5, ST1 = acupuncture points; 5-FU = 5-fluorouracil; DED = dry eye disease; AK = actinic keratoses; Er:YAG = erbium:yttrium-aluminum-garnet; RFM = radiofrequency microneedling; BID = twice daily dosage; Hqol = hyperhidrosis quality of life questionnaire; MASI = melasma area and severity index; MelasQol = melasma quality of life; MSIT = Minor’s starch iodine test

Study ID	Study Design	Population Details	Condition Treated	Microneedling Protocol	Adjunct Therapy (if any)	Comparator / Control	Outcome Measures	Key Results	Adverse Events	Authors' Conclusion
Huang et al., 2024 (China) [7]	RCT, prospective, assessor-blinded, 2-arm	80 patients with moderate-to-severe acne (IGA score 3–4); Fitzpatrick III–IV	Moderate-to-severe acne vulgaris	Single MRF using Unicorn I; 3.5 mm needle, 1–2 pulses/lesion (6–8 W), 3 sessions, 2-week intervals	None	PDT with 5% ALA and red light	IGA score improvement, lesion count (inflammatory and non-inflammatory), satisfaction, VAS pain, adverse events	MRF superior for inflammatory lesions (81% vs 73% reduction); faster response, higher satisfaction; IGA success rates higher in MRF at session 3; non-inflammatory lesions improved similarly in both	MRF had less PIH (10% vs 42%), pain, and acneiform eruptions; all PIH resolved within 3–6 months	MRF is a safe and effective alternative to PDT for acne, with faster efficacy, fewer side effects, and better patient satisfaction [7]
Fatemi Naeini et al., 2015 (Iran) [8]	Sham-controlled, single-blind, intraindividual comparative study	25 patients (32% male, 68% female), mean age 30.2 ± 6.3; Fitzpatrick III–IV; baseline HDSS 3–4	Primary axillary hyperhidrosis	3 sessions of FMR (INFINI™); 3-week intervals; depth 2–3 mm; energy 6–10; 3 passes/session	None	Contralateral sham control (standby device)	HDSS, VAS sweating intensity, patient satisfaction, histopathology	HDSS and VAS scores significantly improved on treated side (HDSS: 1.87 vs 3.38; VAS: 3.92 vs 8.44); 88% had ≥1 score reduction in HDSS; 80% had ≥50% satisfaction	Transient erythema (68%), bleeding (56%), PIH (44%), 1 dropout from transient dysesthesia	FMR is effective and safe for treating PAH; histology showed sweat gland atrophy; minimal side effects [8]
Rummaneethorn and Chalermchai, 2020 (Thailand) [9]	RCT, assessor-blinded, intra-individual split-side	20 female participants; mean age 36.8 ± 9.8 years; diagnosed with primary axillary hyperhidrosis	Primary axillary hyperhidrosis	Fractional microneedle RF (DeAge EX®); 2 sessions, 4 weeks apart; 4 passes/session (depth 3.5 mm and 3.0 mm)	None	Contralateral axilla treated with 50 units BT-A	HDSS, TEWL, DLQI, satisfaction, pain VAS	Both treatments reduced HDSS; BT-A superior at 12 weeks (HDSS: 1.60 vs 2.05, p = 0.0332); better DLQI (p = 0.013) and satisfaction (p = 0.004)	Mild erythema (10%), desquamation (5%), burning (5%) with RF; dryness (5%) with botulinum	BT-A more effective than FMR; FMR still improved outcomes and was well tolerated [9]
Chhabra et al., 2021 (India) [13]	RCT, prospective, hospital-based, single-blind	46 patients (23 per group); age 10–50; 143 vitiligo patches; patch stability ≥1 year	Localized stable vitiligo	1.5 mm Dermapen every 15 days for 4 months (up to 8 sessions)	5-FU 50 mg/mL solution applied post-Mn (Group A)	Mn alone (Group B)	Grading of repigmentation (G0–G4), adverse events	Group A: 48.6% patches showed >75% repigmentation vs 16.9% in Group B (p = 0.0001); response started by 4 weeks in 61.1% (vs 40.8%); better results in trunk and limbs	Common in both: pain (~80%), erythema (~70%), hyperpigmentation (48% vs 26%), ulceration (17% vs 9%)	Mn with 5-FU is safe, simple, and more effective than Mn alone in treating localized vitiligo [13]
Song and Park, 2024 (South Korea) [14]	Assessor-blinded, single-center, parallel RCT	30 patients (15 per group), mean age ~34.3 years, ~57% female	DED	BMA to 5 bilateral facial acupoints (BL2, GB14, TE23, EX-HN5, ST1); 12 sessions over 4 weeks	None	IDA using traditional thumbtack needles	OSDI, VAS, QoL, Schirmer I test (tear production)	Both BMA and IDA significantly improved OSDI, VAS, QoL, and SIT (p < 0.05); no significant difference between groups except higher tear secretion in BMA for left eye (p = 0.025)	No adverse events reported in either group	BMA is as effective and safe as IDA for treating DED; may be preferred for patients allergic to metal needles [14]
Petukhova et al., 2017 (USA) [15]	RCT, single-blind, split-face, 2-arm	33 participants (32 completed); mean age ~63–65; all had ≥3 grade II AK on the face	AK	Microneedle roller (200 μm); 8 passes/side; 10-min or 20-min ALA incubation	ALA + blue light PDT	Sham roller on opposite face side	AK clearance %, TEWL, pain VAS	20-min group: 76% vs 58% clearance (p < 0.01); 10-min group: no significant difference; pain low in both groups	One dropout due to excessive erythema and pain from sun exposure; no moderate/severe pain reported in other participants	20-min ALA incubation with Mn significantly improves AK clearance with minimal pain; similar to 1-hr conventional PDT [15]
Abdel-Hamid et al., 2024 (Egypt) [16]	RCT, 2-arm, parallel, open-label	40 patients with localized stable vitiligo (20 per group); age 10–60; matched for sex, Fitzpatrick type III–V	Vitiligo	Mn using Dermapen (0.25–0.5 mm); every 2 weeks × 6 months	PRP	Comparator: fractional Er:YAG + PRP group	VASI, PGA, repigmentation grade, satisfaction, adverse events	Mn+PRP group: 80% showed repigmentation (20% very good, 10% good, 50% satisfactory); VASI decreased significantly (p = 0.003); 25% reported adverse events	Pain (25%), no serious adverse events; more frequent side effects in Mn group vs Er:YAG group (p = 0.048)	Both Mn+PRP and Er:YAG + PRP induced repigmentation, but Er:YAG + PRP was more effective with fewer side effects and higher satisfaction [16]
Tsai et al., 2025 (Taiwan) [17]	RCT, split-face, vehicle-controlled	30 female participants; mean age 50.3 ± 8.0; Fitzpatrick types III–IV	Refractory melasma	4 sessions of RFM (Sylfirm®) at 1-month intervals; 1.5 mm depth, 2 passes, 25 non-insulated needles	Cysteamine serum (in-office) + home-based cysteamine cream (Group A); placebo or partial protocols in Groups B–D	RFM + placebo serum + vehicle cream (Group D)	mMASI, VISIA imaging (wrinkles, texture, UV spots), patient-reported improvement	Group A had the greatest reduction in mMASI (3.39 → 1.79; p = 0.003) and best VISIA improvements (wrinkles, texture, UV spots); Group D had least improvement	Mostly mild (erythema 26.7%, pain 13.3%, allergic 6.7%); no severe adverse events	RFM combined with in-office and home-based cysteamine is safe and significantly more effective than either alone; promotes melanin reduction and dermal rejuvenation [17]
Kim et al., 2025 (South Korea) [18]	RCT, prospective, double-blinded, split-neck, placebo-controlled	31 subjects (90% female); mean age 49.5 ± 10.4; Fitzpatrick skin types III–IV	Neck skin aging (wrinkles, laxity, elastosis)	2 FMR sessions (GENIUS), 1.5–1.8 mm depth, 20–30 mJ/pin); 4-week interval	Daily topical antioxidant serum (vitamin C, E, ferulic acid; SkinCeuticals®)	Placebo (same base formula without antioxidants)	Fitzpatrick Wrinkle and Elastosis Scale, GAIS, elasticity %, histology (elastin, p16, γ-H2A.X)	Antioxidant side showed superior outcomes: wrinkles ↓29.9% vs ↓18.0%, elasticity ↑12.9% vs ↑2.3%, GAIS (87.5% vs 14.3%); histology: ↑elastin, ↓p16, ↓γ-H2A.X	2 allergic contact dermatitis cases (nickel/cobalt); mild erythema/stinging otherwise	Combining FMR with antioxidants enhances skin rejuvenation clinically and histologically vs FMR alone [18]
Ebrahim and Albalate, 2020 (Egypt) [19]	RCT, 3-arm, randomized controlled	90 patients with localized stable vitiligo (30 per group); age 12–60; ~53% female	Vitiligo	Dermapen (1.5–2 mm); every 2 weeks for up to 12 sessions; occluded topical tacrolimus applied post-procedure for 6 hours	Tacrolimus (0.1% ointment	Group II: Mn alone; Group III: tacrolimus alone (BID x 6 months)	Repigmentation grade (G0–G4), HMB-45 immunostaining, session count, adverse events	Group I (Mn + tacromilus): 76.6% excellent/very good response vs 36.6% (Mn) and 39.9% (Tacromilus); fewer sessions (5 vs 11); highest HMB-45 expression in Group I	Mild pain, erythema, and itching; all transient and tolerable	Mn + tacrolimus superior to either alone in achieving faster, better repigmentation, particularly on extremities [19]
Sadeghzadeh-Bazargan et al., 2024 (Iran) [20]	Blinded, split-face RCT	25 patients (80% female), mean age 35.96 ± 9.23	Atrophic acne scars	3 sessions of Mn (1–1.5 mm depth) at monthly intervals	Topical phenytoin 1% cream (3×/day for 1 week post-Mn on one side)	Mn alone on contralateral face	Pore and spot count/area (Visioface), Goodman and Baron scar grading, patient satisfaction	Both groups improved over time, but the phenytoin side showed greater reduction in acne scar grade and higher satisfaction (80% excellent vs 0%); differences in pore/spot metrics were not statistically significant	None reported	Combining Mn with phenytoin yields superior improvement in scar severity and satisfaction compared to Mn alone [20]
Kim et al., 2013 (South Korea) [21]	Pilot prospective study (single-arm)	20 patients (17 females, 3 males); mean age 30.5 (range 19–46); Fitzpatrick type IV	PAH	2 sessions of FMR (Infini™) at 4-week intervals; 6 passes/session (depths: 3.5 mm, 3.0 mm, 2.5 mm); bipolar RF energy	None	No control group	HDSS, starch-iodine test, TEWL, patient satisfaction, histopathology	HDSS decreased from 3.5 to 1.5 (post 1st) and 1.8 (post 2nd); 75% achieved HDSS 1–2; 70% reported >50% sweat reduction; histology: glandular atrophy	Mild transient swelling, redness, tingling; 2 cases compensatory hyperhidrosis; 1 case transient numbness	FMR is effective and well tolerated for PAH with clinical and histological evidence of sweat gland reduction [21]
Eid et al., 2024 (Egypt) [22]	RCT, split-body, 12-month follow-up	20 patients (85% female), age 18–48; PAH grade 3–4	Primary axillary hyperhidrosis	Fractional microneedle RF (Vivace®), 3.5 mm non-insulated needles; 4 sessions, 3-week intervals; 1 pass/session	None	BT-A (50 IU intradermal injection)	Minor’s test, HDSS, Hqol, patient satisfaction, VAS pain	BT-A showed superior efficacy at 3, 6, 12 months for all outcomes (HDSS, MSIT, QoL, satisfaction); FMR declined after 3 months; BT-A had longer-lasting effects	FMR: more pain (VAS 6.48 vs 4.0), one case itching; BT-A: 2 cases compensatory hyperhidrosis; no serious adverse events	FMR not superior to BT-A; BT-A more effective, less painful, more durable and economical; both were safe [22]
Iraji et al., 2021 (Iran) [23]	RCT, split-lesion, intraindividual	15 patients, 30 lesions (each treated side-by-side); age 18–56; 53% male	Refractory stable vitiligo (limbs)	Mn (Med Amiea revive pen); every 2 weeks for 3 months; depth 0.5 mm adjusted to pinpoint bleeding	Topical pimecrolimus 1% cream (applied immediately post-Mn under occlusion, BID for 3 months)	Contralateral lesion treated with pimecrolimus 1% alone	Repigmentation (0–100%), DLQI, patient satisfaction	Combination group: significant improvement over time (up to 33% good, 6.7% excellent response at 6 months); monotherapy: no significant change	One case of burning with pimecrolimus; no adverse events with Mn	Mn enhances topical pimecrolimus efficacy for stable vitiligo, especially in resistant limb lesions [23]
Kuster Kaminski Arida et al., 2021 (Brazil) [24]	RCT, double-blind, split-face, placebo-controlled	20 women; mean age 42.3 ± 7 years; Fitzpatrick II–V; 60% family history of melasma	Facial melasma	3 monthly sessions of 1.5 mm dermaroller (200 needles); full-face treatment until erythema; topical TA or saline applied to each hemiface (randomized)	TA 50 mg/mL (1 mL)	Placebo (0.9% saline) on contralateral hemiface	Hemi-MASI, Reveal® imaging (pixels), MelasQol, expert and patient evaluation	Hemi-MASI ↓29% (TA) vs ↓22% (control); no significant difference (p = 0.52); similar results in pixel brightness and satisfaction; both improved QoL (↓MelasQol 44.4 → 34.15)	Mild erythema, edema, hematoma; 60% reported temporary darkening lasting median of 5.5 days	Mn + TA showed no added benefit vs Mn + placebo; improvement likely due to Mn + triple topical formula [24]

Protocols varied in depth (0.25-3.5 mm), device (rollers, dermapens, RF-microneedling), session number (2-12), and interval (biweekly to monthly). FMR was common in hyperhidrosis and acne [7,18,22,24], while manual/dermapen microneedling was typical for pigmentary and scarring conditions [13,20,24].

Adjunctive agents included tacrolimus [19], 5-FU [13], pimecrolimus [23], tranexamic acid [24], cysteamine [17], and phenytoin [20], generally producing better outcomes than monotherapy. Some trials incorporated antioxidants or PDT [15,21]. Controls included sham, untreated contralateral sites, placebo, botulinum toxin A, and lasers.

Outcome measures matched the condition: repigmentation scale (vitiligo), hyperhidrosis disease severity scale (HDSS) (hyperhidrosis), investigator’s global assessment (IGA) (acne), modified melasma area and severity index (mMASI) (melasma), wrinkle/elasticity scales (photoaging), and histological/imaging assessments (human melanoma black 45 immunostaining marker (HMB-45), transepidermal water loss (TEWL), VISIA, global aesthetic improvement scale (GAIS)). Combination therapies typically outperformed monotherapy, e.g., microneedling + tacrolimus [19] for repigmentation and microneedle radiofrequency (MRF) over PDT for acne [7].

Quality Assessment

Methodological quality was high (scores 18-24/27, modified Downs and Black). Thirteen studies scored ≥22; only Kim et al. [18] scored 18 due to a pilot, single-arm design. Reporting was strong, with most scoring 10/10; Petukhova et al. [15] and Kim et al. [18] scored 9 and 8, respectively. External validity was consistent (2/3), reflecting moderate generalizability. Internal validity (bias) scores were mostly 5-6/7; confounding control was also strong (5-6/6). Statistical power was adequate in all but Kim et al. [18] (Table 2).

**Table 2:** Quality assessment scores of included studies Reporting: quality of reporting (0–10), External Validity: generalizability of study findings (0–3), Internal Validity - Bias: risk of bias within the study (0–7), Internal Validity - Confounding: control of confounding factors (0–6), Power: statistical power (0–1), Total: overall quality score (0–27)

Study ID	Reporting (0–10)	External Validity (0–3)	Internal Validity - Bias (0–7)	Internal Validity - Confounding (0–6)	Power (0–1)	Total (0–27)
Huang et al., 2024 (China) [7]	10	2	6	5	1	24
Fatemi Naeini et al., 2015 (Iran) [8]	9	2	5	4	1	21
Rummaneethorn and Chalermchai, 2020 [9]	10	2	6	5	1	24
Chhabra et al., 2021 (India) [13]	9	2	5	5	1	22
Song and Park, 2024 (South Korea) [14]	10	2	6	5	1	24
Petukhova et al., 2017 (USA) [15]	9	2	6	5	1	23
Abdel-Hamid et al., 2024 (Egypt) [16]	10	2	6	5	1	24
Tsai et al., 2025 (Taiwan) [17]	10	2	6	5	1	24
Kim et al., 2025 (South Korea) [18]	10	2	6	5	1	24
Ebrahim and Albalate, 2020 (Egypt) [19]	9	2	6	5	1	23
Sadeghzadeh-Bazargan et al., 2024 (Iran) [20]	10	2	6	5	1	24
Kim et al., 2013 (South Korea) [21]	8	2	5	3	0	18
Eid et al., 2024 (Egypt) [22]	10	2	6	5	1	24
Iraji et al., 2021 (Iran) [23]	10	2	6	5	1	24
Kuster Kaminski Arida et al., 2021 (Brazil) [24]	10	2	6	5	1	24

Top-rated studies (24/27) included Rummaneethorn and Chalermchai, Abdel-Hamid et al., Eid et al., Huang et al., Iraji et al., Kim et al., Tsai et al., Kuster Kaminski Arida et al., Song and Park, and Sadeghzadeh-Bazargan et al. [7,9,14,16,17,20-22].

Effect of Intervention

Microneedling-based interventions have demonstrated efficacy across a variety of dermatological conditions, consistently improving clinical outcomes, patient satisfaction, and safety profiles. In photoaging, Kim et al. [21] showed that combining microneedling with topical antioxidants led to a 29.9% reduction in the Fitzpatrick photoaging scale and a 12.9% increase in skin elasticity. These improvements were supported by GAIS assessments and histological findings, including increased elastin expression and decreased markers of cellular senescence such as p16 and γ-H2AX. Minimal transient side effects were reported, indicating favorable tolerability.

For pigmentary disorders such as melasma, studies indicate that microneedling combined with pharmacologic agents enhances efficacy. Tsai et al. [17] found that fractional RF microneedling with cysteamine produced significant mMASI and VISIA score improvements, particularly when paired with home-based cysteamine. Similarly, Kuster Kaminski Arida et al. [24] reported that microneedling with tranexamic acid significantly reduced hemi-melasma area and severity index (hemi-MASI) scores and improved melasma quality of life (MelasQoL) outcomes. Across these studies, transient erythema, edema, and mild local pain were the most common adverse events. Together, these findings suggest that microneedling acts synergistically with antioxidant or depigmenting agents to enhance clinical outcomes.

In conditions not directly involving microneedling, such as dry eye disease, Song and Park [12] demonstrated that biotherapy interventions alone improved ocular surface disease index (OSDI) scores, tear secretion, and quality-of-life parameters without adverse events. This underscores that microneedling’s therapeutic benefits are condition-specific, yet safe alternative interventions exist for other disorders.

Microneedling is also effective for acne and acne scarring. Sadeghzadeh-Bazargan et al. [20] showed that adding phenytoin to microneedling significantly improved acne scar grading compared to microneedling alone (p = 0.001), with higher patient satisfaction and no serious adverse effects. Huang et al. [7] found microneedle RF more effective than PDT in reducing inflammatory lesions (81% vs 73%), achieving higher IGA success rates, and producing greater patient satisfaction, while postinflammatory hyperpigmentation and acneiform eruptions were lower. These results highlight microneedling’s versatility for both scar remodeling and active inflammatory acne, particularly when combined with adjunctive therapies.

In vitiligo, combination therapies with microneedling consistently improved repigmentation and quality-of-life scores. Iraji et al. [23] observed good to excellent repigmentation in 40% of lesions with microneedling plus pimecrolimus and significant DLQI improvement, with no adverse events. Chhabra et al. [13] reported that microneedling plus 5-FU achieved 48.6% excellent repigmentation compared to 16.9% with 5-FU alone, particularly on the trunk and limbs, while mild ulceration and hyperpigmentation were occasionally noted. Comparisons with erbium:yttrium-aluminum-garnet (Er:YAG) laser revealed superior repigmentation and patient satisfaction with laser therapy [16], though microneedling still improved vitiligo area severity index (VASI) scores. Ebrahim and Albalate [19] confirmed that microneedling combined with tacrolimus yielded superior repigmentation (76.6%) compared to monotherapy, supported by histological evidence of melanocyte restoration.

Microneedling has also been explored in hyperhidrosis. While botulinum toxin A demonstrated greater short-term efficacy and patient satisfaction compared to FMR [9,22], studies confirmed that FMR reduces HDSS scores and sweat production meaningfully [8,18]. Histology revealed sweat gland structural changes, and side effects were mostly transient, highlighting FMR as a safe, minimally invasive alternative.

Finally, microneedling can enhance topical therapies in actinic keratoses. Petukhova et al. [15] demonstrated that microneedling-assisted aminolevulinic acid (ALA)-PDT with a 20-minute incubation significantly increased lesion clearance (76% vs 58%; p < 0.01), with manageable pain and increased TEWL, suggesting enhanced drug delivery. A shorter 10-minute incubation did not produce comparable efficacy, emphasizing the importance of procedural optimization.

## Conclusions

Microneedling demonstrates substantial promise as a versatile, minimally invasive therapeutic modality across a broad spectrum of dermatologic conditions, ranging from pigmentary disorders and inflammatory dermatoses to scarring and adnexal dysfunction. Its ability to enhance transdermal delivery of pharmacologic agents such as tranexamic acid, phenytoin, and immunomodulators contributes to synergistic treatment effects and potentially superior clinical outcomes compared to monotherapy. The procedure is generally well tolerated, with adverse effects being mild and transient in most reports, supporting its favorable safety profile. Nevertheless, despite encouraging evidence from multiple randomized controlled trials, heterogeneity in study design, treatment parameters, and outcome measures limits the generalizability of current findings. Future research should prioritize large-scale, standardized, methodologically rigorous trials with extended follow-up to refine procedural protocols, identify predictors of response, and establish the durability of therapeutic benefits.
